# Advances in deformity correction, limb lengthening and reconstruction

**DOI:** 10.1051/sicotj/2018003

**Published:** 2018-04-06

**Authors:** Yasser Elbatrawy

**Affiliations:** Al-Azhar University Faculty of Medicine for girls Cairo, Cairo Egypt

## Abstract

This article summarizes the content of this special issue of the journal about: deformity correction, limb lengthening and reconstruction.

I am honoured to be the guest editor of this special issue of SICOT-J about deformity correction, limb lengthening and reconstruction.

This field of Orthopaedic surgery became a specialty itself in the last 25 years with a high pace in the last 10 years. This is due to the advancement in its technique and the devices that we used from classic Ilizarov device to the I fixator, Orthosuv and the older TSF and hexapod devices that added a lot to the specialty by accurate 6 axes deformity correction.

While some years ago, the only available method for lengthening was utilizing external fixators either circular as in Ilizarov or monolateral as in Wagner or Orthofix LRS, nowadays we have successful available new technique and internal device for lengthening using magnetic intra-medullary nail with remote control that are gaining popularity worldwide among the specialists and the patients as Precice nail which is the only FDA approved internal nail available in the market for lengthening. However, some other nails are available but they are yet to be ap

proved to use in the US market.

In this issue, we are publishing very important articles in the field by 5 main distinguished authors and their associates. The published articles are about a new surgical technique in Radial club hand management or about 3D Printing or about using Precice nail for lengthening and for deformity correction and simultaneous lengthening as well. The last article also is showing the results of a new device for bone grafting in non-union which is the RIA system.

All articles have great illustrations that depict well the prescribed technique.

The first article “*The Paley ulnarization of the carpus with ulnar shortening osteotomy for treatment of radial club hand*” [1] is a milestone in the specialty, as Dror Paley from West Palm Beach, Florida, USA is publishing his new noble modification for his original technique about Ulnarization in radial club hand management. His technique gained more popularity among the specialists worldwide after it was published in 2008.

In his previous original article, he prescribed the value of transferring the hand completely to the ulnar side not just centralizing it as we used to do previously; however, after 9 years and in this article, he modified his original technique by: placing the carpus more on top of the side of the head of the ulna rather than completely on the side of the head of the ulna to protects from the overgrowth of the ulna later on in the life of the patient.

The article describes very well the new technique with illustrations step by step. We see that it is very valuable to publish it, for the specialists to know the recent update in this operation. I appreciate this article and the new modification that shows a broad special experience in the field for the author.

The second important article in our issue is about 3D printing in Orthopedics.

Actually, 3D printing in medicine started years ago however, it started to gain popularity in Orthopedics only in the last few years.

There is an increase in the level of interest in developing uses of 3D modelling and 3D printing in orthopaedic surgery, as demonstrated by a number of recent publications. The new technology of 3D printing has revolutionized and gradually transformed manufacturing across a broad spectrum of industries, and healthcare is not an exception to this. Its popularity in medicine and surgery has grown rapidly over the past several years, and new applications are evolving at an accelerated pace.

The possibility of customized manufacturing using 3D printers has opened new horizons within complex post-traumatic limb reconstruction. One such novel strategy involves patient-specific custom 3D printed titanium truss cages that can be used to address the extremely difficult problem of segmental bone loss often associated with post-traumatic deformities.

In article titled “*Putting 3D modelling and 3D printing into practice: virtual surgery and preoperative planning to reconstruct complex post-traumatic skeletal deformities and defects*”, Dr. Kevin Tetsworth and his colleagues from Australia and USA are exploring the world of 3D printing, which is a revolution in Orthopaedic surgery that will carry a lot of advantages to our patients as well as to the Industry.

I much appreciate this article and the great message that it carries to us as Orthopaedic surgeons [2].

The 3rd article is titled “*PRECICE^®^ magnetically-driven, telescopic, intramedullary lengthening nail: pre-clinical testing and first 30 patients*”. The corresponding author is one of the world’s leaders in this field: Dr. John Herzenberg from Baltimore, Maryland, USA.

He and his associates wrote this interesting article about their first experience with Precice nail and showing honestly its pros and cons by comparing it to other nails available in the market [3].

The 4th article in this issue is also about using the intra-medullary lengthening nail of Precice. However, it is about using the Precice nail for lengthening after acute correction of the deformities around the knee. Dr. Austin T. Fragomen^*^ and Dr. S. Robert Rozbruch from the HSS of New York, USA well describe the surgical technique step by step for acute correction of the deformities in the distal Femur and the Proximal Tibia utilizing mono-lateral fixator before locking and blocking the Precice nail for later lengthening to compensate the lower limb discrepancy associated.

I am sure that the reader will enjoy their article titled “*Lengthening and deformity correction about the knee using a magnetic internal lengthening nail*”, as I personally did [4].

The last article in this issue that is written by Dr. Amr Abdelgawad and his associates from Elpaso, Texas technical university, Texas, USA is really an excellent one showing the results of the RIA system for bone grafting in non-union cases after open fracture.

The article titled “*Bone grafting via reamer-irrigator-aspirator for nonunion of open Gustilo-Anderson type III tibial fractures treated with multiplanar external fixator*” is an interesting article showing the value of RIA system and comparing it to the other classic techniques for bone grafting [5].

I am sure that you will enjoy reading this special issue of SICOT-J about deformity correction, limb lengthening and reconstruction.
